# Self-reported fruit and vegetable intake obtained through a brief telephone interview among users of the *Academia da Saúde* Program: a validation study, Belo Horizonte, Minas Gerais, Brazil, 2016-2017

**DOI:** 10.1590/S2237-96222026v35e20250817.en

**Published:** 2026-06-24

**Authors:** Maria Cecília Ramos de Carvalho, Mariana Souza Lopes, Patrícia Pinheiro de Freitas, Aline Cristine Souza Lopes

**Affiliations:** 1 Universidade Federal de Minas Gerais, Grupo de Pesquisa de Intervenções em Nutrição, Belo Horizonte, MG, Brazil Universidade Federal de Minas Gerais Grupo de Pesquisa de Intervenções em Nutrição Belo Horizonte MG Brazil; 2 Universidade Federal da Paraíba, Departamento de Nutrição, João Pessoa, PB, Brazil Universidade Federal da Paraíba Departamento de Nutrição João Pessoa PB Brazil; 3 Universidade Federal dos Vales do Jequitinhonha e Mucuri, Departamento de Nutrição, Diamantina, MG, Brazil Universidade Federal dos Vales do Jequitinhonha e Mucuri Departamento de Nutrição Diamantina MG Brazil; 4 Universidade Federal de Minas Gerais, Departamento de Nutrição, Belo Horizonte, MG, Brazil Universidade Federal de Minas Gerais Departamento de Nutrição Belo Horizonte MG Brazil

**Keywords:** Nutritional Assessment, Fruit, Vegetables, Eating, Validation Study, Evaluación nutricional, Frutas, Hortalizas, Ingesta de Alimentos, Estudio de Validación

## Abstract

**Objective:**

To assess the validity of fruit and vegetable intake data among users of a health promotion service of the Brazilian Unified Health System (*Sistema Único de Saúde*, SUS).

**Methods:**

Validation study conducted between 2016 and 2017 in the Health Academy Program (*Programa Academia da Saúde*) in Belo Horizonte, Minas Gerais, Brazil. Fruit and vegetable intake was estimated using a brief questionnaire administered in a telephone interview and validated through face-to-face interviews. For statistical analyses of the total sample and the sex-stratified sample, one-sample t-tests, the Concordance Correlation Coefficient (CCC), Bland-Altman plots, and limits of agreement were used.

**Results:**

A total of 441 respondents were included, of whom 378 (85.7%) were women, and 255 (57.8%) were adults. After stratification by sex, the differences between methods were equal to zero. Agreement between the methods was substantial for fruit intake (CCC ranging from 0.632 to 0.696), and low to moderate for vegetable intake (CCC ranging from 0.397 to 0.500). When stratified by education level, the differences were not equal to zero, but there was moderate-to-substantial agreement between the methods for fruit intake (CCC ranging from 0.572 to 0.766) and moderate agreement for vegetable intake (CCC ranging from 0.405 to 0.513). The Bland-Altman plots showed that the differences between the methods were centered around zero and remained stable at higher intake levels.

**Conclusion:**

Data obtained from telephone interviews were valid for estimating fruit and vegetable intake among SUS users.

## Resumen

**Objetivo:**

Verificar la validez de los datos sobre el consumo de frutas y hortalizas entre los usuarios de un servicio de promoción de la salud del Sistema Único de Salud (*Sistema Único de Saúde*, SUS).

**Métodos:**

Estudio de validación realizado entre 2016 y 2017 en el Programa Academia de la Salud (*Programa Academia da Saúde*) de Belo Horizonte, Minas Gerais. El consumo de frutas y hortalizas se estimó mediante un cuestionario breve, aplicado en una entrevista telefónica y validado en una entrevista presencial. Para los análisis estadísticos de la muestra total y de la estratificada por sexo, se utilizaron la prueba t de una muestra, el coeficiente de correlación de concordancia (CCC), los gráficos de Bland-Altman y los límites de concordancia.

**Resultados:**

Se incluyeron 441 participantes, de los cuales 378 (85,7%) eran mujeres y 255 (57,8%) eran adultos. Tras la estratificación por sexo, las diferencias entre los métodos fueron iguales a cero. La concordancia entre los métodos fue sustancial para frutas (CCC entre 0,632 y 0,696) y baja y moderada para hortalizas (CCC entre 0,397 y 0,500). Al estratificar por nivel educativo, las diferencias no fueron iguales a cero, pero se observó una concordancia de moderada a sustancial entre los métodos para frutas (CCC entre 0,572 y 0,766) y de moderada para hortalizas (CCC entre 0,405 y 0,513). Los gráficos de Bland-Altman mostraron que las diferencias entre los métodos se concentraron en torno a cero y se mantuvieron estables en los niveles más altos de consumo.

**Conclusión:**

Los datos obtenidos mediante entrevistas telefónicas resultaron válidos para estimar el consumo de frutas y hortalizas entre los usuarios del Sistema Único de Salud.

**Ethical aspects**

**Table:** This research respected the ethical principles, having obtained the following approval data:

Research Ethics Committee	Opinion number	Certificate of Submission for Ethical Appraisal:	Approval date
Universidade Federal de Minas Gerais	0537.0.203.000-11	0537.0.203.000-11	16/12/2011
Secretaria Municipal de Saúde de Belo Horizonte	0537.0.203.410-11A	0537.0.203.410-11A	22/12/2011
**Registration informed consent** Obtained from all participants prior to data collection.

## Introduction

Adequate intake of unprocessed and minimally processed foods, such as fruits and vegetables, is recommended for health promotion and disease prevention ^1^. However, worldwide and in Brazil, their intake remains below the 400 g per day recommended by the World Health Organization. ^2^. According to the Global Burden of Disease Study, in 2023, 41% of the world’s population had a diet low in fruit, and 26% had a diet low in vegetables. ^3^. In Brazil, in 2023, only 21% of adults reported consuming at least five servings of fruits and vegetables at least five times per week ^1^.

Simple and valid dietary assessment instruments are needed to evaluate fruit and vegetable intake in populations, especially in health services ^1^^),(^^4^^),(^^5^. Such data are needed for longitudinal studies and for the planning, monitoring, and evaluation of policies and interventions, particularly in primary health care ^1^^),(^^4^. However, several challenges are involved in obtaining data on fruit and vegetable intake in health services: the length of questionnaires, the validity of self-reported data, and the lack of standardization regarding the exclusion of starchy roots and tubers from the vegetable group ^4^^)-(^^6^. 

Estimates of fruit and vegetable intake should be based on methods with adequate validity and feasible for use in the routine of health services ^1^^),(^^4^. It is also necessary to use appropriate data collection methods among populations with low educational levels but high telephone coverage ^1^. One example of a lower-cost method is data collection through telephone interviews, which reduces transportation requirements for interviewers and the need for physical space ^1^^),(^^4^^),(^^5^. 

Data on fruit and vegetable intake obtained through telephone interviews were validated in the Behavioral Risk Factor Surveillance System in the United States between 1991 and 2000, and in the Surveillance System for Risk and Protective Factors for Chronic Diseases by Telephone Survey (*Vigilância de Fatores de Risco e Proteção para Doenças Crônicas por Inquérito Telefônico*, Vigitel) in Brazil in 2005 ^7^^),(^^8^. However, the samples in these studies included adults from the general population and residents of state capitals, respectively, but did not investigate health service users.

Furthermore, there is no gold standard for estimating fruit and vegetable intake; a valid instrument is required for use as the reference method. ^4^^),(^^5^. When a reference method is used, only the relative validity of the test method can be assessed ^9^. In the context of assessing relative validity, a brief face-to-face instrument was validated to estimate daily fruit and vegetable intake among users of a health promotion service in Belo Horizonte between 2013 and 2014. These data showed moderate correlation with 24-hour dietary recall data, used as the reference method: 0.427 for fruit, 0.283 for vegetables, and 0.361 for fruits and vegetables ^9^. However, the instrument has not been validated for use in telephone interviews. In this context, the objective of the study was to assess the validity of fruit and vegetable intake data among users of a health promotion service within the Brazilian Unified Health System (*Sistema Único de Saúde*, SUS). 

## Methods

### Study design

This validation study was conducted from March 2016 to February 2017. It follows the Guidelines for Reporting Reliability and Agreement Studies (GRRAS) for agreement analyses and the Strengthening the Reporting of Observational Studies in Epidemiology (STROBE) guidelines for cross-sectional studies ^10^^),(^^11^. 

### Setting

The study was carried out within the Health Academy Program (*Programa Academia da Saúde*) in Belo Horizonte, Minas Gerais, Brazil. The municipality, the sixth largest in Brazil, has implemented the Program since 2006 ^12^. It is a health promotion service integrated into primary health care that aims to encourage healthy lifestyles through weekly supervised physical exercise sessions, health promotion actions, food and nutrition activities, health education, and integrative and complementary practices ^12^. The Program units were preferably implemented in census tracts with a higher health vulnerability index. This index served as an eligibility criterion for the study and was estimated from socioeconomic and environmental data. It ranges from 0 to 1 and is classified as low, medium, high, or very high ^13^^)-(^^15^. 

### Participants

This study analyzed data from participants of a randomized controlled community trial conducted with a representative sample of the Health Academy Program in Belo Horizonte. 

The sample size calculation considered the 50 program units existing in Belo Horizonte at the end of 2012. The inclusion criteria were:

- morning operation (preferred service operating hours);
- located in census tracts with medium, high, or very high health vulnerability (priority areas for program implementation); and
- no participation in nutritional intervention studies in the previous two years.

Six units located in areas with low health vulnerability and two that had recently participated in a nutritional intervention were excluded. 42 units were eligible ^14^.

Simple random sampling, stratified by the municipality’s nine administrative regions, was conducted. Among the eligible units in each region, two were randomly selected for inclusion in the study. The two units in each region were randomized into intervention or control groups. The nutritional intervention aimed at promoting fruit and vegetable intake, whose data are not addressed in this study, was described in a protocol ^13^. The final sample consisted of 18 program units. This sample was representative of units located in areas with medium, high, and very high health vulnerability, with 95% confidence and an error margin of less than 1.4%, calculated after the study was conducted ^13^.

In each sampled unit, eligible participants were adult users (aged 20 years or older) who had attended the service at least once in the previous month. Pregnant women and individuals with cognitive impairment that prevented questionnaire completion were excluded. Pregnant women were excluded because one of the objectives of the larger study was to assess body weight changes, and results could be biased by weight changes during pregnancy ^15^. 

Baseline data collection was conducted between 2013 and 2014, with a total of 3,414 participants. Participants from the baseline who were alive between 2016 and 2017 were eligible for follow-up through telephone interviews. 

### Variables

The following baseline and telephone interview data were used: sex (female; male); age (in years, categorized as adults: up to 59 years; older adults: ≥60 years); education (in years: up to 8 years; 9 to 11 years; 12 years or more); monthly per capita income (in BRL, categorized according to the 50th percentile); marital status (married/stable union; divorced; single; widowed); self-reported diagnosis of hypertension, dyslipidemia, and diabetes; height measured at baseline; and self-reported weight collected by telephone. 

Self-reported weight was validated in a previous study, which reported concordance correlation coefficients of 0.94 or higher between measured and self-reported values. Weight was used to assess nutritional status according to the Body Mass Index [BMI=weight (kg)/height (m)^2^]. Nutritional status was classified as underweight (BMI<18.5 kg/m²); normal weight (BMI between 18.5 kg/m² and 24.9 kg/m²); overweight (BMI between 25.0 and 29.9 kg/m²); and obesity (BMI≥30.0 kg/m²) ^15^. 

Fruit and vegetable intake was estimated using a brief questionnaire administered via telephone interviews (test method). Validation was performed using the same questionnaire applied in face-to-face interviews (reference method). 

Using the brief questionnaire, validated for face-to-face ^9^ administration, the usual frequency of fruit and vegetable intake and the number of servings consumed on a typical day were estimated. Servings consumed on a typical day were recorded using household measures (units, slices, cups, spoons, etc.). Household measures were converted into a standardized 80-gram serving, as recommended by the World Health Organization ^2^^),(^^9^. 

As a serving of fruit, one unit, slice, or cup was considered. As a serving of raw leafy greens, four tablespoons, two serving spoons, one dessert plate, or half a meal plate were considered. As a serving of cooked or sautéed leafy greens and other vegetables, two tablespoons, one serving spoon, half a dessert plate, or one-quarter of a meal plate were considered ^2^^),(^^9^. Juices, smoothies, roots, and tubers were excluded due to their distinct nutritional profiles. Participants were instructed not to consider these foods when reporting their fruit and vegetable intake ^9^.

Participants separately reported the frequency of fruit and vegetable intake: never (less than once per month or not consumed), rarely (1-3 times/month), 1-2 times/week, 3-4 times/week, 5-6 times/week, every day (including Saturdays and Sundays) ^9^.

For each method, two variables were created to express the average daily intake of fruits and vegetables, respectively. The variables were created only for individuals who reported consuming at least one of the food groups at least once per month. The midpoint of each frequency category represented intake frequency, and the variables were constructed according to the following formulas: 

Fruit and vegetable intake at least once per month but less than once per week:

Daily intake=(Servings consumed on a typical day*frequency of intake)/30

Fruit and vegetable intake at least once per week: 

Daily intake=(Servings consumed on a typical day*frequency of intake)/7

For each food group, the number of servings was multiplied by 80 to estimate the mean daily intake in grams. ^9^. Differences between intake estimates from the two methods (g/day) were calculated by subtracting values from face-to-face interviews from those from telephone interviews. 

### Data sources and measurement

Landline and/or mobile phone numbers were obtained from the baseline database, from records of Health Academy Program units, or from telephone directories. For each available phone number, four call attempts were made Monday through Saturday, in the morning, afternoon, and evening. For participants who agreed to participate in data collection, the telephone interview was conducted at the same time or at a scheduled time. If contact was not established after four attempts, the team searched again for telephone numbers in program unit records and telephone directories. Four additional attempts were made for each newly identified number^15^.

Participants selected for the validation study were contacted again and invited to participate in face-to-face data collection. For those who agreed to participate in the validation study, face-to-face data collection was conducted at the participant’s affiliated program unit. Those who refused to participate were replaced with other users selected from the same Health Academy Program unit and stratum. At least two participants per stratum were sought to minimize selection bias ^15^.

### Bias

Field supervisors reviewed paper-based questionnaires used at baseline before data entry into the system. During the telephone interviews, four interviewers were trained and supervised to collect data. The supervisor was responsible for ensuring the standardization of data collection, auditing a random sample of questionnaires, and conducting repeat training sessions as needed. The databases underwent consistency analyses after data entry. Procedure handbooks were made available to interviewers during data collection ^15^. 

Participants for the validation study were randomly selected after the telephone interviews were completed at each Health Academy Program unit. This approach avoided bias in data collection through telephone interviews, based on participants’ selection status for the validation study. 

Face-to-face data collection was scheduled to avoid intervals that were too short (<5 days), during which participants might recall their responses from the telephone interview, or too long (>60 days), during which participants might have changed their fruit and vegetable intake. During face-to-face data collection, interviewers did not have access to data from the telephone interviews.

### Study size

Data were collected from 2,370 participants through telephone interviews, and 15.0% of respondents were randomly selected for this validation study. This sample size was considered sufficient based on validation studies of self-reported body weight ^15^. A list of eligible respondents was created for each unit, stratified by sex (female; male), age group (20-29 years; 30-59 years; 60 years or older), attendance at the program in the previous month (present; absent), and nutritional status (underweight or normal weight; overweight or obesity). A random number generator (“sorteador.com.br”) was used to select two or more participants per stratum for the validation study. Individuals were excluded if they refused or could not be reached by telephone after four attempts ^15^ (Figure 1).

**Figure 1:**
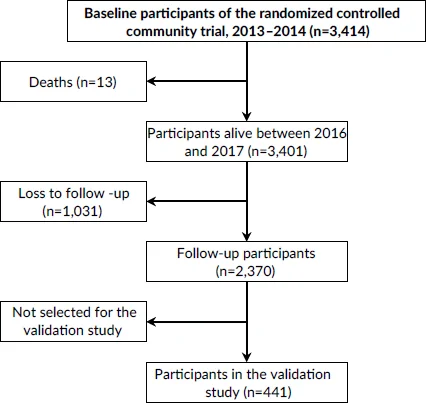
Sampling process stages

The trial sample was representative of program units located in territories with medium, high, or very high health vulnerability, with a 95% confidence level and a margin of error of less than 1.4% ^13^. For the validation study, statistical power was calculated after data collection had been completed, based on the sample effectively evaluated. This calculation compared sociodemographic characteristics between participants in the validation study and all program participants, yielding a statistical power of 99.0% ^15^. 

### Statistical methods

Data were entered into Microsoft Access by trained and supervised undergraduate and graduate students. Consistency, descriptive, and multivariate analyses were performed using Stata 14.2 (College Station, Texas, United States of America). Categorical variables were described using frequencies and 95% confidence intervals (95%CI). The normality of continuous variables was assessed using the Shapiro-Wilk test, and these variables were described using means and 95%CI. A significance level of 5% was adopted for all analyses. 

Various statistical analyses were used to assess the validity of fruit and vegetable intake data collected via telephone interviews. Validity analyses were performed for the total sample and stratified by sex and educational level. One-sample Student’s t-tests were used to determine whether the mean differences between methods were equal to zero. Agreement between daily fruit and vegetable intake data obtained by telephone and face-to-face interviews was analyzed using the Concordance Correlation Coefficient (CCC) ^16^. Landis and Koch cutoff points were applied to classify agreement as very poor (CCC≤0.20), poor (0.21≤CCC≤0.40), moderate (0.41≤CCC≤ 0.60), substantial (0.61≤CCC≤0.80), and almost perfect (CCC>0.80) ^17^. The distribution of differences and the 95% limits of agreement between the results obtained by the two methods were analyzed using Bland-Altman plots ^18^. Sensitivity analyses were performed after excluding participants with outlying data, whose fruit and vegetable intake exceeded the 95th percentile of the respective food group. Intake above the 95th percentile was considered outlying because fruit and vegetable intake is a desirable behavior and may be overestimated, which could lead to lower agreement with data from face-to-face interviews ^4^. 

## Results

Among the 441 respondents included in the validation study, 378 (85.7%) were women, 255 (57.8%) were adults, 183 (41.5%) were overweight, and 267 (60.5%) had completed up to 8 years of schooling (Table 1). 

**Table 1:** Absolute and relative frequencies and 95% confidence intervals (95%CI) of the characteristics of participants in the validation study. Belo Horizonte, 2013-2017 (n=441)

Characteristics	Participants in the validation study (n=441)	
	n (%)	95%CI
Sex (%)		
Female	378 (85.7)	82.1; 88.7
Male	63 (14.3)	11.3; 17.9
**Age group (%)**		
Adult	255 (57.8)	53.1; 62.4
Older adult	186 (42.2)	37.6; 46.9
**Educational level (%)**		
Up to 8 years of schooling	267 (60.5)	55.9; 65.0
9-11 years of schooling	136 (30.9)	26.7; 35.3
12 years of schooling or more	38 (8.6)	6.3; 11.6
**Monthly per capita income (%)** ^a^		
Up to the 50th percentile	213 (52.3)	47.5; 57.2
Above the 50th percentile	194 (47.7)	42.8; 52.5
**Marital status (%)**		
Married/stable union	286 (64.9)	60.3; 69.2
Divorced	35 (7.9)	5.7; 10.9
Single	54 (12.2)	9.5; 15.7
Widowed	66 (15.0)	11.9; 18.6
High blood pressure (%)	227 (51.5)	46.8; 56.1
Dyslipidemia (%)	197 (45.2)	40.5; 49.9
Diabetes (%)	77 (17.5)	14.2; 21.3
**Nutritional status (%)**		
Underweight	20 (4.5)	2.9; 6.9
Normal weight	157 (35.6)	31.3; 40.2
Overweight	183 (41.5)	37.0; 46.2
Obesity	81 (18.4)	15.0; 22.3

^a^407 participants had valid data on monthly per capita income.

Fruit intake reported in the telephone interviews was 145.9 g/day, and vegetable intake was 172.5 g/day. Fruit intake reported in the face-to-face interviews was 154.4 g/day, and vegetable intake was 169.4 g/day. The mean differences between methods were different from zero for fruit intake in the total sample (p-value 0.039) and in the subgroup with up to 8 years of schooling (p-value 0.009). The differences were equal to zero after stratification by sex (p-value 0.098 for women; p-value 0.129 for men) and in the groups with 9 to 11 years (p-value 0.212) or 12 or more years of schooling (p-value 0.170) (Table 2). 

**Table 2:** Means, concordance correlation coefficients (CCC), differences between methods, and 95% confidence intervals (95%CI) in the total sample and according to sex and schooling for estimated fruit and vegetable intake obtained through telephone interviews and face-to-face interviews. Belo Horizonte, 2013-2017 (n=441)

Food group and sample stratification	Intake according to test and reference methods: mean (95%CI)		Lin’s CCC (95%CI)	Classification of Lin’s CCC^a^	Difference between methods: mean (95%CI)^b^	p-value^c^
	Telephone	Face-to-face				
Fruits						
Total sample	145.9	154.4	0.642	Substantial	-8.1	**0.039**
(n=436)	(137.1; 154.7)	(145.2; 163.7)	(0.587; 0.697)		(-15.7; -0.4)	
Female	149.3	156.6	0.632	Substantial	-7.1	0.098
(n=375)	(139.8; 158.8)	(146.6; 166.7)	(0.571; 0.692)		(-15.5; 1.3)	
Male	125.6	140.9	0.696	Substantial	-14.1	0.129
(n=61)	(102.9; 148.3)	(116.5; 165.3)	(0.567; 0.826)		(-32.3; 4.2)	
Up to 8 years of schooling	141.6	156.4	0.572	Moderate	-14.7	**0.009**
(n=266)	(130.0; 153.2)	(144.2; 168.6)	(0.492; 0.653)		(-25.7; -3.8)	
9 to 11 years of schooling	150.2	144.8	0.764	Substantial	6.8	0.212
(n=136)	(135.0; 165.5)	(128.8; 160.9)	(0.693; 0.835)		(-3.9; 17.6)	
12 years of schooling or more	160.8	175.0	0.766	Substantial	-14.3	0.170
(n=38)	(131.9; 189.6)	(142.3; 207.8)	(0.635; 0.897)		(-35.0; 6.4)	
**Vegetables**						
Total sample	172.5	169.4	0.415	Moderate	3.2	0.507
(n=432)	(164.2; 180.8)	(160.3; 178.6)	(0.337; 0.493)		(-6.3; 12.6)	
Female	175.0	172.2	0.397	Poor	3.3	0.536
(n=371)	(166.2; 183.8)	(162.2; 182.2)	(0.312; 0.482)		(-7.1; 13.6)	
Male	157.8	152.6	0.500	Moderate	2.8	0.814
(n=61)	(133.1; 182.5)	(129.2; 176.0)	(0.309; 0.691)		(-21.1; 26.7)	
Up to 8 years of schooling	172.2	169.6	0.405	Moderate	1.5	0.821
(n=267)	(161.7; 182.6)	(156.8; 182.5)	(0.306; 0.505)		(-11.3; 14.3)	
9 to 11 years of schooling	174.5	171.0	0.417	Moderate	6.0	0.484
(n=136)	(157.8; 191.1)	(156.5; 185.4)	(0.278; 0.556)		(-10.8; 22.7)	
12 years of schooling or more	168.2	162.9	0.513	Moderate	5.3	0.652
(n=38)	(146.3; 190.2)	(137.3; 188.5)	(0.277; 0.749)		(-18.2; 28.7)	

^a^very poor (CCC≤0.20); poor (0.21≤CCC≤0.40); moderate (0.41≤CCC≤0.60); substantial (0.61≤CCC≤0.80); almost perfect (CCC>0.80); ^b^Difference=telephone-face-to-face; ^c^One-sample t-test, H0: difference=0.

In the total sample, the CCC indicated substantial agreement for fruit intake (0.642) and moderate agreement for vegetable intake (0.415). After stratification by sex, agreement was substantial for fruit intake (0.632 to 0.696) and ranged from low (0.397) to moderate (0.500) for vegetable intake. After stratification by education, agreement ranged from moderate to substantial for fruit intake (CCC ranging from 0.572 to 0.766) and remained moderate for vegetable intake (CCC ranging from 0.405 to 0.513) (Table 2).

The Bland-Altman plots showed that the differences between methods were distributed around zero. The estimated 95% limits of agreement between the methods were similar across food groups, sexes, and educational-level categories. The differences between methods did not appear to increase or decrease with increasing intake (Figure 2). 

**Figure 2:**
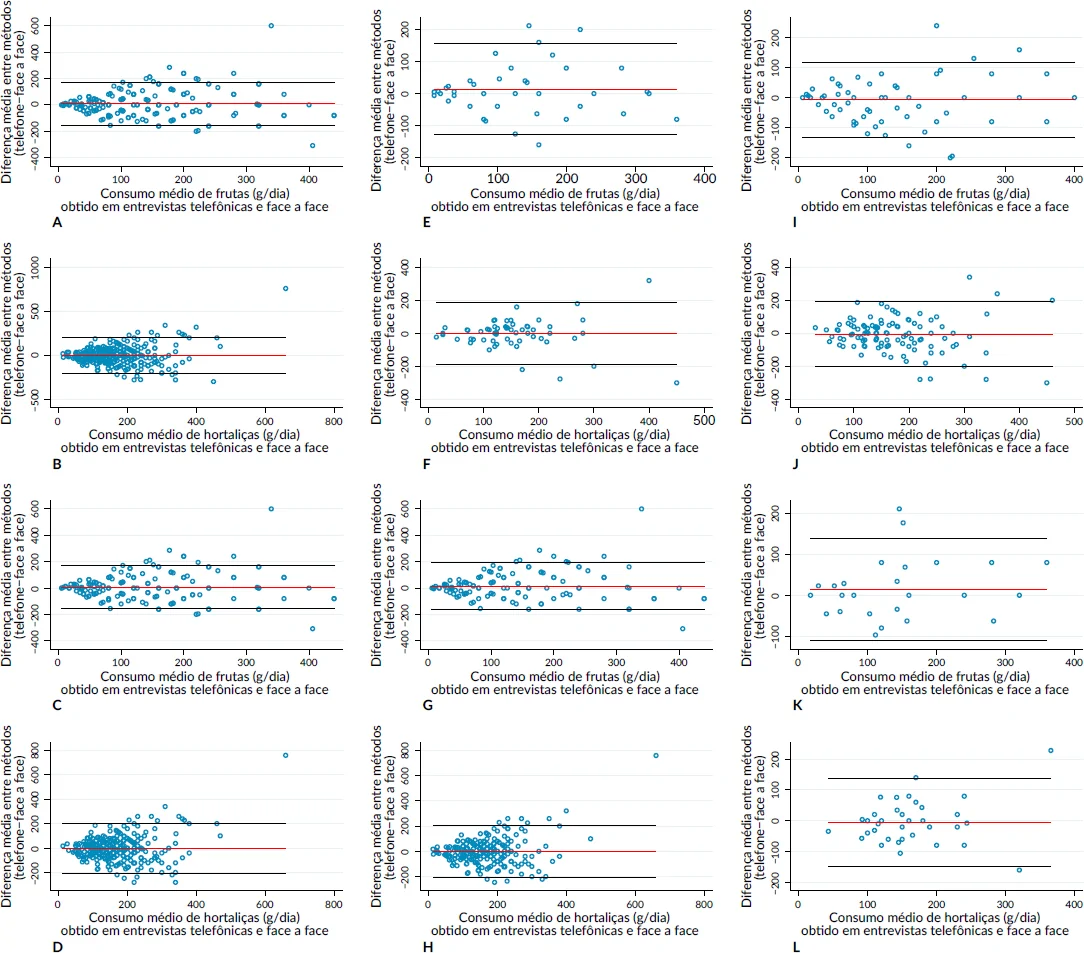
Bland-Altman plots of differences between fruit and vegetable intake obtained through telephone and face-to-face interviews, according to sex and educational level. Belo Horizonte, 2013-2017 (n=441) Total sample (A), (B); female (C), (D); male (E), (F); up to 8 years of schooling (G), (H); 9 to 11 years of schooling (I), (J); 12 years of schooling or more (K), (L).

Exclusion of outliers (8 participants for fruit intake and 17 for vegetable intake) did not change the estimated intakes or the differences between methods (Table 3). The CCC for fruit intake in the total sample and in sex-stratified analyses also indicated substantial agreement. In the analysis stratified by educational level, the CCC for fruit intake among participants with a lower educational level increased from 0.572 (moderate agreement) to 0.623 (substantial agreement). The CCC for vegetable intake among men increased from 0.500 (moderate agreement) to 0.621 (substantial agreement) (Table 3).

**Table 3:** Means, Lin’s concordance correlation coefficients (CCC), differences between methods, and 95% confidence intervals (95%CI) in the total sample and according sex and schooling for estimated fruit and vegetable intake obtained through telephone interviews and face-to-face interviews among participants with fruit and vegetable intake below the 95th percentile. Belo Horizonte, 2013-2017 (n=428)

Food group and sample stratification	Intake according to test and reference methods: mean (95%CI)		Lin’s CCC (95%CI)	Classification of Lin’s CCC^a^	Difference between methods: mean (95% CI)^b^	p-value^c^
	Telephone	Face-to-face				
Fruits						
Total sample	139.0	146.9	0.629	Substantial	-7.4	0.039
(n=428)	(131.1; 146.8)	(138.5; 155.4)	(0.572; 0.687)		(-14.3; -0.4)	
Female	141.6	148.0	0.624	Substantial	-5.9	0.113
(n=367)	(133.2; 150.0)	(138.9; 157.0)	(0.560; 0.687)		(-13.5; 1.6)	
Male	123.0	140.9	0.658	Substantial	-16.0	0.091
(n=61)	(101.7; 144.3)	(116.5; 165.3)	(0.514; 0.802)		(-34.6; 2.6)	
Up to 8 years of schooling	142.4	154.9	0.623	Substantial	-12.5	0.015
(n=263)	(130.7; 154.1)	(143.2; 166.6)	(0.549; 0.697)		(-22.6; -2.4)	
9 to 11 years of schooling	151.6	144.8	0.764	Substantial	6.8	0.212
(n=134)	(136.3; 167.0)	(128.8; 160.9)	(0.693; 0.835)		(-3.9; 17.6)	
12 years of schooling or more	160.8	175.0	0.766	Substantial	-14.3	0.170
(n=38)	(131.9; 189.6)	(142.3; 207.8)	(0.635; 0.897)		(-35.0; 6.4)	
**Vegetables**						
Total sample	161.2	156.9	0.419	Moderate	4.7	0.216
(n=415)	(154.3; 168.2)	(150.2; 163.6)	(0.337; 0.501)		(-2.8; 12.2)	
Female	164.7	159.4	0.382	Poor	5.6	0.190
(n=356)	(157.1; 172.3)	(152.2; 166.5)	(0.290; 0.473)		(-2.8; 13.9)	
Male	140.3	142.2	0.621	Substantial	-0.5	0.945
(n=59)	(123.5; 157.1)	(123.9; 160.4)	(0.457; 0.786)		(-15.9; 14.9)	
Up to 8 years of schooling	170.7	166.3	0.436	Moderate	4.4	0.449
(n=259)	(160.2; 181.2)	(155.2; 177.4)	(0.337; 0.534)		(-7.0; 15.9)	
9 to 11 years of schooling	176.9	171.0	0.417	Moderate	6.0	0.484
(n=134)	(160.4; 193.4)	(156.5; 185.4)	(0.278; 0.556)		(-10.8; 22.7)	
12 years of schooling or more	168.2	162.9	0.513	Moderate	5.3	0.652
(n=38)	(146.3; 190.0)	(137.3; 188.5)	(0.277; 0.749)		(-18.2; 28.7)	

^a^very poor (CCC≤0.20); poor (0.21≤CCC≤0.40); moderate (0.41≤CCC≤0.60); substantial (0.61≤CCC≤0.80); almost perfect (CCC>0.80); ^b^Difference=telephone-face-to-face. ^c^One-sample t-test, H0: difference=0.

## Discussion

No significant differences were identified between fruit and vegetable intake data obtained using a brief questionnaire administered through telephone interviews (test method) and those collected through face-to-face interviews (reference method). Regarding fruit intake, sex may have confounded the analysis of the full sample, suggesting a difference that was no longer significant after stratification. Participants with a lower educational level showed a non-zero error between methods, but agreement remained moderate. 

One limitation to consider is that fruit and vegetable intake was self-reported and may have been overestimated. However, the reference method had been previously validated in the study population and compared with 24-hour dietary recall data ^9^. Selecting 15% of respondents for the validation study could have reduced the study’s statistical power; however, a power calculation conducted after data collection indicated 99% power in the sample. The time elapsed since data collection may also be considered a limitation. However, given the sample size, extensive analyses of data consistency were required. The validation analyses could only be completed in 2021.

The concordance correlation coefficients identified in this study were similar to those reported in studies conducted in the United States ^8^^),(^^19^^),(^^20^. The estimated correlations ranged from low (0.29) to moderate (0.56) for fruit intake, from moderate (0.47) to substantial (0.63) for vegetable intake, and from low (0.22) to substantial (0.77) for combined fruit and vegetable intake ^8^^),(^^19^^),(^^20^. These studies were conducted between 1993 and 2015 using samples from the general population ^8^, groups of mothers, adults, and older adults ^19^, and Black women who were overweight ^20^. In addition, the limits of agreement according to the Bland-Altman plots were acceptable ^20^. The association between lower educational level and greater error in reporting fruit intake was similar to findings among adults with a low educational level in the United States in 2022 ^21^. In that study, a difference between methods was observed for fruit intake, but not for vegetable intake. Likewise, agreement between methods remained moderate despite the difference identified between methods ^21^.

In validation studies of fruit and vegetable intake data obtained through telephone interviews with Brazilian samples composed of adults with landline phones residing in Belo Horizonte (2009), São Paulo (2005), and Belém (2009), 24-hour dietary recall was selected as the reference method to assess fruit and vegetable intake. The prevalence of regular fruit and vegetable intake (≥5 days/week) was compared across methods, with sensitivity ranging from 35% to 89% and specificity from 29% to 84% ^7^^),(^^23^^),(^^24^.

Also in the United States, between 1993 and 2015, the validity of fruit and vegetable intake data obtained through telephone interviews was investigated, and Spearman’s or Pearson’s correlation coefficients were reported ^8^^),(^^19^^),(^^20^. However, Spearman’s and Pearson’s correlation coefficients are not appropriate measures of validity. Instead, concordance correlation coefficients and Bland-Altman plots are appropriate for adequately assessing agreement between data obtained using different methods ^16^^),(^^18^^),(^^22^.

Overall, the results of this study were consistent with those of previous studies ^8^^),(^^19^^)-(^^21^: the concordance correlation coefficients were substantial for fruit intake and moderate for vegetable intake. In addition, the Bland-Altman plots and their limits of agreement indicated greater validity of the test method compared with the results of a study involving a sample of low-income women ^20^. 

Strengths of this study include the various procedures used to ensure and control data quality, as well as the use of appropriate analyses for assessing validity (Lin’s Concordance Correlation Coefficients and Bland-Altman plots). 

In conclusion, fruit and vegetable intake, estimated using a brief instrument administered via telephone interviews, provides sufficiently valid data to estimate intake among users of a SUS health promotion service.

## Data Availability

The contents of the database are available upon request to the corresponding author.
