# Supplementary material for: Self-reported fruit and vegetable intake obtained through a brief telephone interview among users of the Academia da Saúde Program: a validation study, Belo Horizonte, Minas Gerais, Brazil, 2016-2017
Source: Epidemiol Serv Saude. 2026 Jun 24;35:e20250817. doi: 10.1590/S2237-96222026v35e20250817.en (PMC13686308; doi:10.1590/S2237-96222026v35e20250817.en)
Supplement: Translation

# Translation: Consumo autorreferido de frutas e hortaliças obtido por entrevista telefônica breve entre usuários do Programa Academia da Saúde: estudo de validação, Belo Horizonte, Minas Gerais, 2016-2017

**DOI:** 10.1590/S2237-96222026v35e20250817.pt

**Authors:** Maria Cecília Ramos de Carvalho, Mariana Souza Lopes, Patrícia Pinheiro de Freitas, Aline Cristine Souza Lopes

**Affiliations:** 1 Universidade Federal de Minas Gerais, Grupo de Pesquisa de Intervenções em Nutrição, Belo Horizonte, MG, Brasil; 2 Universidade Federal da Paraíba, Departamento de Nutrição, João Pessoa, PB, Brasil; 3 Universidade Federal dos Vales do Jequitinhonha e Mucuri, Departamento de Nutrição, Diamantina, MG, Brasil; 4 Universidade Federal de Minas Gerais, Departamento de Nutrição, Belo Horizonte, MG, Brasil

**Keywords:** Avaliação Nutricional, Frutas, Hortaliças, Ingestão de alimentos, Estudo de Validação

## Abstract

**Objetivo:**

Verificar a validade de dados de consumo de frutas e hortaliças entre usuários de serviço de promoção da saúde do Sistema Único de Saúde.

**Métodos:**

Estudo de validação conduzido entre 2016 e 2017 no Programa Academia da Saúde de Belo Horizonte, Minas Gerais. O consumo de frutas e hortaliças foi estimado por questionário breve aplicado em entrevista telefônica e validado face a face. Para análises estatísticas da amostra total e estratificada por sexo, foram utilizados teste t de uma amostra, Coeficiente de Correlação de Concordância (CCC), gráficos de Bland-Altman e limites de concordância.

**Resultados:**

Foram incluídos 441 respondentes, sendo 378 (85,7%) mulheres, e 255 (57,8%) adultos. Após estratificação por sexo, as diferenças entre métodos foram iguais a zero. A concordância entre os métodos foi substancial para frutas (CCC entre 0,632 e 0,696), e entre baixa e moderada para hortaliças (CCC entre 0,397 e 0,500). Ao estratificar por escolaridade, as diferenças não foram iguais a zero, mas houve concordância de moderada a substancial entre os métodos para frutas (CCC entre 0,572 e 0,766), e moderada para hortaliças (CCC entre 0,405 e 0,513). Os gráficos de Bland-Altman mostraram que as diferenças entre métodos se distribuíram em torno de zero, sendo estáveis nos maiores níveis de consumo.

**Conclusão:**

Os dados de entrevistas telefônicas foram válidos para estimar o consumo de frutas e hortaliças entre usuários do Sistema Único de Saúde.

**Aspectos éticos**

**Table:** Esta pesquisa respeitou os princípios éticos, obtendo os seguintes dados de aprovação

Comitê de ética em pesquisa	Número do parecer	Certificado de apresentação de apreciação ética	Data de aprovação
Universidade Federal de Minas Gerais	0537.0.203.000-11	0537.0.203.000-11	16/12/2011
Secretaria Municipal de Saúde de Belo Horizonte	0537.0.203.410-11A	0537.0.203.410-11A	22/12/2011
**Registro do consentimento livre e esclarecido** Obtido de todos os participantes antes da coleta dos dados.

## Introdução

O consumo adequado de alimentos in natura e minimamente processados, como frutas e hortaliças, é recomendado para promoção da saúde e prevenção de doenças ^1^. Porém, seu consumo no mundo e no Brasil é inferior aos 400 g ao dia recomendados pela Organização Mundial da Saúde ^2^. Segundo o estudo Global Burden of Disease, em 2023, 41% da população mundial tinha uma dieta pobre em frutas, e 26% apresentava uma dieta pobre em hortaliças ^3^. No Brasil, em 2023, apenas 21% dos adultos relataram consumir pelo menos cinco porções de frutas e hortaliças cinco ou mais vezes por semana ^1^.

Inquéritos alimentares de uso simples e válidos são necessários para avaliar o consumo de frutas e hortaliças em populações, especialmente em serviços de saúde ^1^^),(^^4^^),(^^5^. Tais dados são necessários para estudos longitudinais, planejamento, monitoramento e avaliação de políticas e intervenções, sobretudo na atenção primária à saúde ^1^^),(^^4^. Entretanto, há diversos desafios na obtenção de dados do consumo de frutas e hortaliças nos serviços de saúde: a extensão dos questionários, a validade dos dados autorreferidos e a ausência de padronização quanto à não inclusão de raízes e tubérculos como hortaliças ^4^^)-(^^6^. 

Estimativas do consumo de frutas e hortaliças devem basear-se em métodos com validade adequada, e viáveis para uso na rotina de serviços de saúde ^1^^),(^^4^. Também é necessário utilizar métodos apropriados de coleta de dados entre populações com baixa escolaridade, mas com alta cobertura de telefonia ^1^. Um exemplo de método com menor custo consiste na obtenção de dados por entrevista telefônica, o que reduz o deslocamento de entrevistadores e a necessidade de espaço físico ^1^^),(^^4^^),(^^5^. 

Dados de consumo de frutas e hortaliças obtidos por entrevista telefônica foram validados no Behavioral Risk Factor Surveillance System nos Estados Unidos, entre 1991 e 2000, e no Sistema de Vigilância de Fatores de Risco e Proteção para Doenças Crônicas por Inquérito Telefônico (VIGITEL) no Brasil, em 2005 ^7^^),(^^8^. Entretanto, as amostras desses estudos incluem adultos da população em geral ou residentes nas capitais, respectivamente, não investigando usuários de serviços de saúde.

Ademais, para estimar o consumo de frutas e hortaliças inexiste um padrão-ouro; é necessário um instrumento válido a ser utilizado como método de referência ^4^^),(^^5^. Quando um método de referência é utilizado, permite-se avaliar apenas a validade relativa do método de teste ^9^. No contexto de avaliação da validade relativa, um instrumento breve de aplicação face a face foi validado para estimar o consumo diário de frutas e hortaliças entre usuários de serviço de promoção da saúde em Belo Horizonte, entre 2013 e 2014. Esses dados apresentaram correlação moderada com os dados de recordatório alimentar 24 horas, utilizado como método de referência: 0,427 para frutas, 0,283 para hortaliças, e 0,361 para frutas e hortaliças ^9^. Porém, o instrumento não foi validado para uso em entrevistas telefônicas. Nesse sentido, o objetivo do estudo foi verificar a validade de dados de consumo de frutas e hortaliças entre usuários de serviço de promoção da saúde do Sistema Único de Saúde. 

## Métodos

### Delineamento do estudo

Este estudo de validação foi realizado de março de 2016 a fevereiro de 2017. Ele segue as Diretrizes para Relato de Estudos de Confiabilidade e Concordância (Guidelines for Reporting Reliability and Agreement Studies, GRRAS) para análises de concordância e o Fortalecimento do Relato de Estudos Observacionais em Epidemiologia (Strengthening the Reporting of Observational studies in Epidemiology, STROBE) para estudos transversais ^10^^),(^^11^. 

### Contexto

O estudo foi realizado no Programa Academia da Saúde de Belo Horizonte, Minas Gerais, Brasil. O município, sexto maior do Brasil, conta com o Programa desde 2006 ^12^. Trata-se de um serviço de promoção da saúde inserido na atenção primária à saúde que objetiva estimular modos saudáveis de vida por meio de sessões semanais supervisionadas de exercícios físicos, ações de promoção da saúde, alimentação e nutrição, educação em saúde e práticas integrativas e complementares ^12^. As unidades do Programa foram implementadas preferencialmente em setores censitários com maior índice de vulnerabilidade à saúde. Tal índice foi utilizado como critério de elegibilidade para o estudo, sendo estimado a partir de dados socioeconômicos e ambientais. Varia de 0 a 1, com classificação baixo, médio, elevado, ou muito elevado ^13^^)-(^^15^. 

### Participantes

Este estudo analisou dados de participantes de ensaio comunitário controlado randomizado conduzido com amostra representativa do Programa Academia da Saúde de Belo Horizonte. 

O cálculo amostral considerou as 50 unidades do programa existentes em Belo Horizonte no momento da amostragem, no final de 2012. Os critérios de inclusão foram:

- funcionamento matutino (horário preferencial de funcionamento do serviço);
- estar localizada em setor censitário com média, elevada ou muito elevada vulnerabilidade à saúde (áreas prioritárias de implementação do programa); e
- não ter participado de estudos de intervenção nutricional nos dois anos anteriores.

Foram excluídas seis unidades em áreas com baixa vulnerabilidade à saúde e duas que haviam participado de intervenção nutricional recente. Ficaram elegíveis 42 unidades ^14^.

Foi realizada amostragem aleatória simples, estratificada pelas nove regionais administrativas do município. Entre as unidades elegíveis por regional, foram sorteadas duas para inclusão no estudo. As duas unidades de cada regional foram randomizadas em intervenção ou controle. A intervenção nutricional de incentivo ao consumo de frutas e hortaliças, cujos dados não são abordados neste estudo, foi descrita em protocolo ^13^. A amostra final foi composta por 18 unidades do programa. Essa amostra foi representativa das unidades em áreas com vulnerabilidade à saúde média, elevada e muito elevada, com 95% de confiança e erro menor que 1,4%, calculados a posteriori ^13^.

Em cada unidade amostrada, foram elegíveis os usuários adultos (com 20 ou mais anos de idade) que tivessem comparecido pelo menos uma vez ao serviço no mês anterior. Foram excluídas gestantes e pessoas com deficiência cognitiva que impossibilitasse de responder ao questionário. As gestantes foram excluídas, pois um dos objetivos do estudo maior era avaliar a evolução do peso corporal; os resultados poderiam ser enviesados devido às alterações de peso na gestação ^15^. 

A coleta de dados da linha de base foi realizada entre 2013 e 2014, totalizando 3.414 participantes. Os participantes da linha de base que estivessem vivos de 2016 a 2017 foram elegíveis para reavaliação por entrevista telefônica. 

### Variáveis

Os seguintes dados da linha de base e da entrevista telefônica foram utilizados: sexo (feminino; masculino); idade (em anos, categorizada em adultos: até 59 anos; idosos≥60 anos); escolaridade (em anos: até 8 anos; 9 a 11 anos; 12 anos ou mais); renda per capita mensal (em Reais, categorizada conforme o percentil 50); estado civil (casado/união consensual; divorciado; solteiro; viúvo); diagnóstico autorreferido de hipertensão arterial, dislipidemia e diabetes; altura aferida na linha de base; peso autorreferido na ligação telefônica. 

O peso autorreferido foi validado em estudo prévio que identificou coeficientes de correlação de concordância iguais ou superiores a 0,94 entre medidas aferidas e autorreferidas. O peso foi utilizado para avaliação do estado nutricional conforme o Índice de Massa Corporal [IMC=peso (kg)/altura (m)^2^]. O estado nutricional foi classificado em baixo peso (IMC<18,5 kg/m²); eutrofia (IMC entre 18,5 kg/m² e 24,9 kg/m²); sobrepeso (IMC entre 25,0 e 29,9 kg/m²), e obesidade (IMC≥30,0 kg/m²) ^15^. 

O consumo de frutas e hortaliças foi estimado por meio de questionário breve aplicado em ligações telefônicas (método de teste). A validação foi realizada a partir do mesmo questionário aplicado em entrevista face a face (método de referência). 

Com o questionário breve, validado para uso face a face ^9^, estimaram-se a frequência habitual de consumo de frutas e hortaliças e a quantidade de porções consumidas em um dia comum. As porções consumidas em um dia comum foram registradas em medidas caseiras (unidades, fatias, xícaras, colheres etc). As medidas caseiras foram convertidas em porções padronizadas de 80 gramas, conforme preconizado pela Organização Mundial da Saúde ^2^^),(^^9^. 

Como porção de frutas, considerou-se uma unidade, fatia ou xícara de chá. Como porção de verduras cruas, consideraram-se quatro colheres de sopa, duas colheres de servir, um prato de sobremesa ou ½ prato de refeição. Como porção de verduras cozidas ou refogadas e de legumes, consideraram-se duas colheres de sopa, uma colher de servir, ½ prato de sobremesa ou ¼ de prato de refeição ^2^^),(^^9^. Sucos, vitaminas, raízes e tubérculos não foram incluídos, tendo em vista suas diferentes características nutricionais. Os participantes foram orientados a não considerar esses alimentos ao relatar seu consumo de frutas e hortaliças ^9^.

Os participantes relataram, separadamente, a frequência de consumo de frutas e de hortaliças: nunca (menos de uma vez por mês ou não consome), quase nunca (1-3 x/mês), 1-2 x/semana, 3-4 x/semana, 5-6 x/semana, todos os dias (incluindo sábados e domingos) ^9^.

Para cada método, foram criadas duas variáveis para expressar o consumo médio diário de frutas e de hortaliças, respectivamente. As variáveis foram criadas apenas para indivíduos que relataram consumir um ou ambos os grupos pelo menos uma vez por mês. A frequência de consumo foi representada pelo ponto médio da categoria de frequência, e as variáveis foram criadas conforme as seguintes fórmulas: 

Consumo de frutas e hortaliças no mínimo uma vez por mês, mas menos que uma vez por semana:

Consumo diário=(porções consumidas em um dia comum * frequência de consumo)/30

Consumo de frutas e hortaliças no mínimo uma vez por semana: 

Consumo diário=(porções consumidas em um dia comum * frequência de consumo)/7

Para cada grupo de alimentos, o número de porções foi multiplicado por 80 para se estimar o consumo médio diário em gramas ^9^. As diferenças entre o consumo estimado pelos dois métodos (g/dia) foram calculadas subtraindo-se os valores obtidos na coleta de dados face a face dos valores obtidos nas entrevistas telefônicas. 

### Fontes de dados e mensuração

Números de telefone fixo e/ou celular foram obtidos no banco de dados da linha de base, nos registros das unidades do Programa Academia da Saúde, ou em listas telefônicas. Para cada número de telefone disponível, foram realizadas quatro tentativas de ligação entre segunda-feira e sábado pela manhã, tarde e noite. Para os participantes que concordaram em participar da coleta de dados, a entrevista telefônica foi conduzida no mesmo momento ou agendada. Em caso de insucesso nas quatro tentativas, a equipe realizou novas buscas por números de telefone nos registros das unidades do programa e em listas telefônicas. Quatro novas tentativas foram realizadas para cada novo número encontrado ^15^.

Os participantes sorteados para o estudo de validação foram contactados novamente e convidados para a coleta de dados face a face. Para aqueles que concordaram em participar do estudo de validação, a coleta de dados foi realizada na unidade do programa de vínculo do participante. Aqueles que se recusaram a participar foram substituídos por outros usuários sorteados na mesma unidade do Programa Academia da Saúde e no mesmo estrato. Procurou-se obter, no mínimo, dois participantes por estrato, minimizando o viés de seleção ^15^.

### Viés

Os questionários em papel, na linha de base, foram verificados pelas supervisoras de campo antes da entrada de dados no sistema. Nas ligações telefônicas, quatro entrevistadores foram treinados e supervisionados para as coletas de dados. A supervisora foi responsável por garantir a padronização da coleta de dados, auditar uma amostra aleatória dos questionários, e repetir treinamentos quando necessário. Os bancos de dados passaram por análises de consistência após a digitação. Manuais dos procedimentos foram disponibilizados para os entrevistadores durante a coleta de dados ^15^. 

O sorteio de participantes para o estudo de validação foi realizado após a finalização das entrevistas telefônicas de cada unidade do Programa Academia da Saúde. Assim, evitou-se viés na coleta de dados por entrevistas telefônicas conforme o status de seleção dos participantes para o estudo de validação. 

A coleta de dados face a face foi agendada de modo a evitar intervalos muito curtos (<5 dias), em que o participante pudesse se lembrar das respostas da entrevista telefônica, ou muito longos (>60 dias), em que o participante pudesse ter mudado seu consumo de frutas e hortaliças. Durante a coleta de dados face a face, os entrevistadores não tiveram acesso aos dados das entrevistas telefônicas.

### Tamanho do estudo

Dados de 2.370 participantes foram coletados por entrevista telefônica, sendo selecionados aleatoriamente 15,0% dos respondentes para este estudo de validação. Identificou-se que esse tamanho amostral seria suficiente com base em estudos de validação do peso corporal autorreferido ^15^. Criou-se uma lista de respondentes elegíveis por unidade, estratificada por sexo (feminino; masculino), faixa etária (20-29 anos; 30-59 anos; 60 anos ou mais), presença no programa no mês anterior (presente; ausente) e estado nutricional (baixo peso ou eutrofia; sobrepeso ou obesidade). Um gerador de números aleatórios (“sorteador.com.br”) foi utilizado para selecionar dois ou mais participantes por estrato para o estudo de validação. Os indivíduos foram excluídos em caso de recusa ou impossibilidade de contato telefônico depois de quatro tentativas ^15^ (Figura 1).

**Figura 1:** Etapas do processo amostral

A amostra do ensaio foi representativa das unidades do programa localizadas em territórios de média, elevada ou muito elevada vulnerabilidade à saúde, com um nível de confiança de 95% e um erro menor que 1,4% ^13^. Para o estudo de validação, o cálculo de poder amostral foi realizado a posteriori, após a coleta de dados da validação, de acordo com a amostra efetivamente avaliada. Esse cálculo levou em consideração uma comparação de características sociodemográficas dos participantes do estudo de validação com o total de participantes do programa, identificando poder estatístico de 99,0% ^15^. 

### Métodos estatísticos

Os dados foram digitados no programa Microsoft Access por estudantes de graduação e pós-graduação, treinados e supervisionados. Análises de consistência, descritivas e multivariadas foram realizadas no programa Stata 14.2 (College Station, Texas, United States of America). As variáveis categóricas foram descritas por frequências e intervalos de confiança de 95% (IC95%). As variáveis contínuas tiveram sua normalidade verificada pelo teste Shapiro-Wilk, e foram descritas pelas médias e IC95%. Considerou-se em todas as análises um nível de significância de 5%. 

Diferentes análises estatísticas foram utilizadas para avaliar a validade dos dados de consumo de frutas e hortaliças obtidos por entrevistas telefônicas. As análises de validade foram realizadas com a amostra total e estratificadas por sexo e escolaridade. Testes t de Student de uma amostra foram utilizados para identificar se as diferenças médias entre os métodos eram iguais a zero. A concordância entre os dados de consumo diário de frutas e hortaliças obtidos por telefone e face a face foi analisada com uso do coeficiente de correlação de concordância (CCC) ^16^. Os pontos de corte de Landis e Koch foram aplicados para classificar a concordância em: muito baixa (CCC≤0,20); baixa (0,21≤CCC≤0,40); moderada (0,41≤CCC≤0,60); substancial (0,61≤CCC≤0,80); quase perfeita (CCC>0,80) ^17^. A distribuição das diferenças e dos limites de concordância de 95% entre os resultados dos dois métodos foi analisada por gráficos de Bland-Altman ^18^. Análises de sensibilidade foram realizadas após a exclusão de participantes com dados discrepantes, cujo consumo de frutas e hortaliças excedia o percentil 95 do respectivo grupo de alimentos. Considerou-se discrepante o consumo acima do percentil 95, dado que o consumo de frutas e hortaliças é um hábito desejável e passível de superestimação, o que poderia levar a uma menor concordância com os dados obtidos face a face ^4^. 

## Resultados

Entre os 441 respondentes do estudo de validação, 378 (85,7%) eram mulheres, 255 (57,8%) adultos, 183 (41,5%) apresentavam sobrepeso e 267 (60,5%) tinham até 8 anos de escolaridade (Tabela 1). 

**Tabela 1:** Frequências absolutas, relativas e intervalos de confiança de 95% (IC95%) das características dos participantes do estudo de validação. Belo Horizonte, 2013-2017 (n=441)

Características	Participantes do estudo de validação (n=441)	
	n (%)	IC95%
Sexo (%)		
Feminino	378 (85,7)	82,1; 88,7
Masculino	63 (14,3)	11,3; 17,9
**Faixa etária (%)**		
Adulto	255 (57,8)	53,1; 62,4
Idoso	186 (42,2)	37,6; 46,9
**Escolaridade (%)**		
Até 8 anos	267 (60,5)	55,9; 65,0
9 a 11 anos	136 (30,9)	26,7; 35,3
12 anos ou mais	38 (8,6)	6,3; 11,6
**Renda mensal per capita (%)** ^a^		
Até o percentil 50	213 (52,3)	47,5; 57,2
Acima do percentil 50	194 (47,7)	42,8; 52,5
**Estado civil (%)**		
Casado/União consensual	286 (64,9)	60,3; 69,2
Divorciado	35 (7,9)	5,7; 10,9
Solteiro	54 (12,2)	9,5; 15,7
Viúvo	66 (15,0)	11,9; 18,6
Hipertensão arterial (%)	227 (51,5)	46,8; 56,1
Dislipidemia (%)	197 (45,2)	40,5; 49,9
Diabetes (%)	77 (17,5)	14,2; 21,3
**Estado nutricional (%)**		
Baixo peso	20 (4,5)	2,9; 6,9
Eutrofia	157 (35,6)	31,3; 40,2
Sobrepeso	183 (41,5)	37,0; 46,2
Obesidade	81 (18,4)	15,0; 22,3

^a^407 participantes com dados válidos de renda mensal per capita.

O consumo relatado por entrevista telefônica foi de 145,9 g de frutas/dia e de 172,5 g de hortaliças/dia. O consumo relatado nas entrevistas face a face foi de 154,4 g de frutas/dia e de 169,4 g de hortaliças/dia. As diferenças médias entre os métodos foram diferentes de zero para o consumo de frutas na amostra total (p-valor 0,039) e no subgrupo com até 8 anos de escolaridade (p-valor 0,009). As diferenças foram iguais a zero após estratificação por sexo (p-valor 0,098 no sexo feminino; p-valor 0,129 no sexo masculino) e nos grupos com 9 a 11 (p-valor 0,212) ou 12 ou mais anos de escolaridade (p-valor 0,170) (Tabela 2). 

**Tabela 2:** Médias, coeficientes de correlação de concordância (CCC), diferenças entre os métodos e intervalos de confiança de 95% (IC95%) na amostra total e segundo sexo e escolaridade do consumo estimado de frutas e hortaliças por entrevistas telefônicas e entrevistas face a face. Belo Horizonte, 2013-2017 (n=441)

Grupo de alimentos e estratificação da amostra	Consumo segundo método de teste e de referência: média (IC95%)		CCC de Lin (IC95%)	Classificação do CCC de Lin^a^	Diferença entre métodos: média (IC95%)^b^	p-valor^c^
	Telefone	Face a face				
**Frutas**						
Amostra total	145,9	154,4	0,642	Substancial	-8,1	0,039
(n=436)	(137,1; 154,7)	(145,2; 163,7)	(0,587; 0,697)		(-15,7; -0,4)	
Sexo feminino	149,3	156,6	0,632	Substancial	-7,1	0,098
(n=375)	(139,8; 158,8)	(146,6; 166,7)	(0,571; 0,692)		(-15,5; 1,3)	
Sexo masculino	125,6	140,9	0,696	Substancial	-14,1	0,129
(n=61)	(102,9; 148,3)	(116,5; 165,3)	(0,567; 0,826)		(-32,3; 4,2)	
Até 8 anos de estudo	141,6	156,4	0,572	Moderada	-14,7	0,009
(n=266)	(130,0; 153,2)	(144,2; 168,6)	(0,492; 0,653)		(-25,7; -3,8)	
9 a 11 anos de estudo	150,2	144,8	0,764	Substancial	6,8	0,212
(n=136)	(135,0; 165,5)	(128,8; 160,9)	(0,693; 0,835)		(-3,9; 17,6)	
12 anos de estudo ou mais	160,8	175,0	0,766	Substancial	-14,3	0,170
(n=38)	(131,9; 189,6)	(142,3; 207,8)	(0,635; 0,897)		(-35,0; 6,4)	
**Hortaliças**						
Amostra total	172,5	169,4	0,415	Moderada	3,2	0,507
(n=432)	(164,2; 180,8)	(160,3; 178,6)	(0,337; 0,493)		(-6,3; 12,6)	
Sexo feminino	175,0	172,2	0,397	Baixa	3,3	0,536
(n=371)	(166,2; 183,8)	(162,2; 182,2)	(0,312; 0,482)		(-7,1; 13,6)	
Sexo masculino	157,8	152,6	0,500	Moderada	2,8	0,814
(n=61)	(133,1; 182,5)	(129,2; 176,0)	(0,309; 0,691)		(-21,1; 26,7)	
Até 8 anos de estudo	172,2	169,6	0,405	Moderada	1,5	0,821
(n=267)	(161,7; 182,6)	(156,8; 182,5)	(0,306; 0,505)		(-11,3; 14,3)	
9 a 11 anos de estudo	174,5	171,0	0,417	Moderada	6,0	0,484
(n=136)	(157,8; 191,1)	(156,5; 185,4)	(0,278; 0,556)		(-10,8; 22,7)	
12 anos de estudo ou mais	168,2	162,9	0,513	Moderada	5,3	0,652
(n=38)	(146,3; 190,2)	(137,3; 188,5)	(0,277; 0,749)		(-18,2; 28,7)	

^a^muito baixa (CCC≤0,20); baixa (0,21≤CCC≤0,40); moderada (0,41≤CCC≤0,60); substancial (0,61≤CCC≤0,80); quase perfeita (CCC>0,80); ^b^Diferença=Telefone-face a face; ^c^Teste t de uma amostra, H0: diferença=0.

Na amostra total, o CCC apresentou concordância substancial para o consumo de frutas (0,642) e moderada para hortaliças (0,415). Após estratificação por sexo, a concordância foi substancial para frutas (0,632 a 0,696) e de baixa (0,397) a moderada (0,500) para hortaliças. Após a estratificação por escolaridade, a concordância variou entre moderada e substancial para frutas (CCC entre 0,572 e 0,766), e permaneceu moderada para hortaliças (CCC entre 0,405 e 0,513) (Tabela 2).

Os gráficos de Bland-Altman revelaram que as diferenças entre métodos distribuíram-se em torno de zero. Os limites de concordância de 95% estimados entre os métodos foram similares entre os grupos de alimentos, sexos e categorias de escolaridade. As diferenças entre métodos não pareceram aumentar ou diminuir com o aumento do consumo (Figura 2).

**Figura 2:** Gráficos de Bland-Altman das diferenças entre o consumo de frutas e hortaliças obtido por entrevistas telefônicas e face a face, segundo o sexo e a escolaridade. Belo Horizonte, 2013-2017 (n=441) Amostra total (A), (B); sexo feminino (C), (D); sexo masculino (E), (F); até 8 anos de estudo (G), (H); 9 a 11 anos de estudo (I), (J); 12 anos de estudo ou mais (K), (L).

A exclusão dos dados discrepantes (8 participantes para o consumo de frutas e 17 para o consumo de hortaliças) não alterou o consumo estimado ou as diferenças entre métodos (Tabela 3). Os CCC para o consumo de frutas na amostra total e estratificada por sexo também foram substanciais. Na estratificação por escolaridade, o CCC para o consumo de frutas entre participantes com menor escolaridade passou de 0,572 (concordância moderada) para 0,623 (substancial). O CCC do consumo de hortaliças para o sexo masculino passou de 0,500 (moderada) para 0,621 (substancial) (Tabela 3).

**Tabela 3:** Médias, coeficientes de correlação de concordância (CCC), diferenças entre os métodos e intervalos de confiança de 95% (IC95%) na amostra total e segundo sexo e escolaridade do consumo estimado de frutas e hortaliças por entrevistas telefônicas e entrevistas face a face entre participantes com consumo de frutas e hortaliças abaixo do percentil 95. Belo Horizonte, 2013-2017 (n=428)

Grupo de alimentos e estratificação da amostra	Consumo segundo método de teste e de referência: média (IC95%)		CCC de Lin (IC95%)	Classificação do CCC de Lin^a^	Diferença entre métodos: média (IC95%)^b^	p-valor^c^
	Telefone	Face a face				
Frutas						
Amostra total	139,0	146,9	0,629	Substancial	-7,4	**0,039**
(n=428)	(131,1; 146,8)	(138,5; 155,4)	(0,572; 0,687)		(-14,3; -0,4)	
Sexo feminino	141,6	148,0	0,624	Substancial	-5,9	0,113
(n=367)	(133,2; 150,0)	(138,9; 157,0)	(0,560; 0,687)		(-13,5; 1,6)	
Sexo masculino	123,0	140,9	0,658	Substancial	-16,0	0,091
(n=61)	(101,7; 144,3)	(116,5; 165,3)	(0,514; 0,802)		(-34,6; 2,6)	
Até 8 anos de estudo	142,4	154,9	0,623	Substancial	-12,5	**0,015**
(n=263)	(130,7; 154,1)	(143,2; 166,6)	(0,549; 0,697)		(-22,6; -2,4)	
9 a 11 anos de estudo	151,6	144,8	0,764	Substancial	6,8	0,212
(n=134)	(136,3; 167,0)	(128,8; 160,9)	(0,693; 0,835)		(-3,9; 17,6)	
12 anos de estudo ou mais	160,8	175,0	0,766	Substancial	-14,3	0,170
(n=38)	(131,9; 189,6)	(142,3; 207,8)	(0,635; 0,897)		(-35,0; 6.4)	
**Hortaliças**						
Amostra total	161,2	156,9	0,419	Moderada	4,7	0,216
(n=415)	(154,3; 168,2)	(150,2; 163,6)	(0,337; 0,501)		(-2,8; 12,2)	
Sexo feminino	164,7	159,4	0,382	Baixa	5,6	0,190
(n=356)	(157,1; 172,3)	(152,2; 166,5)	(0,290; 0,473)		(-2,8; 13,9)	
Sexo masculino	140,3	142,2	0,621	Substancial	-0,5	0,945
(n=59)	(123,5; 157,1)	(123,9; 160,4)	(0,457; 0,786)		(-15,9; 14,9)	
Até 8 anos de estudo	170,7	166,3	0,436	Moderada	4,4	0,449
(n=259)	(160,2; 181,2)	(155,2; 177,4)	(0,337; 0,534)		(-7,0; 15,9)	
9 a 11 anos de estudo	176,9	171,0	0,417	Moderada	6,0	0,484
(n=134)	(160,4; 193,4)	(156,5; 185,4)	(0,278; 0,556)		(-10,8; 22,7)	
12 anos de estudo ou mais	168,2	162,9	0,513	Moderada	5,3	0,652
(n=38)	(146,3; 190,0)	(137,3; 188,5)	(0,277; 0,749)		(-18,2; 28,7)	

^a^muito baixa (CCC≤0,20); baixa (0,21≤CCC≤0,40); moderada (0,41≤CCC≤0,60); substancial (0,61≤CCC≤0,80); quase perfeita (CCC>0,80); ^b^Diferença=Telefone-face a face; ^c^Teste t de uma amostra, H0: diferença=0.

## Discussão

Não foram identificadas diferenças significativas entre dados de consumo de frutas e hortaliças obtidos por questionário breve aplicado por entrevista telefônica (método de teste) e coletados em entrevista face a face (método de referência). Quanto ao consumo de frutas, o sexo pode ter sido um confundidor na análise da amostra completa, acusando uma diferença que deixou de ser significativa após estratificação. Participantes com menor escolaridade apresentaram erro diferente de zero entre os métodos, mas mantiveram concordância moderada. 

Uma limitação deve ser considerada: o consumo de frutas e hortaliças foi autorreferido, e pode ter sido superestimado. Porém, o método de referência foi previamente validado com a população do estudo, tendo sido comparado ao recordatório alimentar de 24 horas ^9^. A seleção de 15% dos respondentes para o estudo de validação poderia reduzir o poder amostral do estudo, porém, o cálculo a posteriori identificou um poder de 99% na amostra. O tempo decorrido desde a coleta dos dados também pode ser uma limitação. Entretanto, considerando o tamanho amostral, foi necessário realizar exaustivas análises de consistência dos dados. Somente em 2021 foi possível finalizar as análises de validação.

Os coeficientes de correlação de concordância identificados neste estudo foram similares aos coeficientes de correlação de estudos realizados nos Estados Unidos ^8^^),(^^19^^),(^^20^. As correlações estimadas variaram de baixa (0,29) a moderada (0,56) para o consumo de frutas, de moderada (0,47) a substancial (0,63) para o consumo de hortaliças, e de baixa (0,22) a substancial (0,77) para o consumo de frutas e hortaliças em conjunto ^8^^),(^^19^^),(^^20^. Tais estudos foram realizados entre 1993 e 2015, com amostras da população em geral ^8^, ou de grupos de mães, adultos e idosos ^19^, ou de mulheres negras com excesso de peso ^20^. Além disso, os limites de concordância de acordo com os gráficos de Bland-Altman foram aceitáveis ^20^. A associação entre menor escolaridade e maior erro no relato do consumo de frutas foi similar ao resultado encontrado entre adultos de baixa escolaridade nos Estados Unidos em 2022 ^21^. No referido estudo, houve diferença entre métodos para o consumo de frutas, mas não para o consumo de hortaliças. Da mesma forma, a concordância entre métodos permaneceu moderada mesmo com a diferença identificada entre métodos ^21^.

Em validação de dados de consumo de frutas e hortaliças obtidos por entrevistas telefônicas com amostras brasileiras, compostas por adultos com linhas telefônicas fixas e residentes em Belo Horizonte (2009), São Paulo (2005) e Belém (2009), o recordatório alimentar 24 horas foi escolhido como método de referência para avaliar o consumo de frutas e hortaliças. As prevalências do consumo regular de frutas e hortaliças (≥5 dias/semana) foram comparadas entre os métodos, e os resultados variaram de 35% a 89% para a sensibilidade, e de 29% a 84% para a especificidade ^7^^),(^^23^^),(^^24^.

Ainda nos Estados Unidos, entre 1993 e 2015, investigou-se a validade de dados de consumo de frutas e hortaliças obtidos por entrevistas telefônicas, reportando-se coeficientes de correlação de Spearman ou de Pearson ^8^^),(^^19^^),(^^20^. Entretanto, coeficientes de correlação de Spearman ou Pearson não configuram medidas adequadas de validade. Em vez disso, é adequado utilizar coeficientes de correlação de concordância e gráficos de Bland-Altman para avaliar adequadamente a concordância entre dados obtidos por diferentes métodos ^16^^),(^^18^^),(^^22^.

Em geral, os resultados deste estudo apontaram para a mesma direção dos estudos anteriores ^8^^),(^^19^^)-(^^21^: os valores dos coeficientes de correlação de concordância foram substanciais para o consumo de frutas, e moderados para o consumo de hortaliças. Além disso, os gráficos de Bland-Altman e seus limites de concordância mostraram a maior validade do método de teste em comparação aos resultados de um estudo com amostra de mulheres de baixa renda ^20^. 

Potencialidades deste estudo incluem os diversos procedimentos empregados para a garantia e controle de qualidade dos dados, e o uso de análises apropriadas para as medidas de validade (Coeficientes de Correlação de Concordância de Lin e gráficos de Bland-Altman). 

Conclui-se que o consumo de frutas e hortaliças estimado por instrumento breve aplicado em entrevistas telefônicas gera dados suficientemente válidos para estimar o consumo de frutas e hortaliças entre usuários de serviço de promoção da saúde do SUS.

## Data Availability

O conteúdo do banco de dados está disponível mediante solicitação ao autor correspondente.
